# A new species of *Thibaudia* (Ericaceae, Vaccinieae) from the Cordillera del Cóndor in Ecuador

**DOI:** 10.3897/BDJ.13.e157044

**Published:** 2025-07-25

**Authors:** Marco M. Jiménez, James L. Luteyn, J. R. Kuethe, Darío García, Nadia Lapo-González, Henry X. Garzón-Suárez, Gabriel A. Iturralde

**Affiliations:** 1 Grupo de Investigación en Medio Ambiente y Salud (BIOMAS), Ingeniería en Agroindustria, Facultad de Ingenierías y Ciencias Aplicadas, Universidad de Las Américas, vía a Nayón, Quito, Ecuador Grupo de Investigación en Medio Ambiente y Salud (BIOMAS), Ingeniería en Agroindustria, Facultad de Ingenierías y Ciencias Aplicadas, Universidad de Las Américas, vía a Nayón Quito Ecuador; 2 Institute of Systematic Botany, New York Botanic Garden, Bronx, New York, United States of America Institute of Systematic Botany, New York Botanic Garden Bronx, New York United States of America; 3 School of Envinronment, University of Auckland, Auckland, New Zealand School of Envinronment, University of Auckland Auckland New Zealand; 4 Macleania Berries, Vereda Mancilla, Facatativá, Colombia Macleania Berries, Vereda Mancilla Facatativá Colombia; 5 Floplaya, Compañía de flores y plantas Yantzaza S.A., El Pangui, Ecuador Floplaya, Compañía de flores y plantas Yantzaza S.A. El Pangui Ecuador; 6 Grupo Científico Calaway Dodson: Investigación y Conservación de Orquídeas del Ecuador, Quito, Ecuador Grupo Científico Calaway Dodson: Investigación y Conservación de Orquídeas del Ecuador Quito Ecuador; 7 Jungle Dave’s Science Foundation, San Juan Bosco, Ecuador Jungle Dave’s Science Foundation San Juan Bosco Ecuador; 8 Herbario HUTPL, Departamento de Ciencias Biológicas, Universidad Técnica Particular de Loja, San Cayetano Alto s/n 11-01-608, Loja, Ecuador Herbario HUTPL, Departamento de Ciencias Biológicas, Universidad Técnica Particular de Loja, San Cayetano Alto s/n 11-01-608 Loja Ecuador

**Keywords:** Andean Tepui, Neotropics, new species, northern Andes, rainforest, Zamora Chinchipe

## Abstract

**Background:**

The neotropical genus *Thibaudia*Ruiz & Pav. ex J.St.-Hil. (Ericaceae), comprises about 70–75 species and is distributed from Honduras in Central America, through the central Andes south to Bolivia, and eastward to Suriname and Brazil in South America. Ecuador is one of the countries with the greatest diversity of the genus, including 18 species, 12 of which are endemic. Within southeastern Ecuador, the Cordillera del Cóndor is a region characterized by exceptionally high levels of plant endemism, largely due to its unique geology. Recent botanical explorations across this area have led to the discovery of several new species in recent years. Among those was a peculiar species of *Thibaudia*that is distinct by having exclusively cauliflorous inflorescences in which the pedicel is articulated with the calyx and the filaments are connate. This species is here described and illustrated as *Thibaudiashagmiana sp. nov.*

**New information:**

A new species, *Thibaudiashagmiana*, is described from the Cordillera del Cóndor in eastern Ecuador. It is distinguished by having a scrambling habit, provided with lignotubers, lanceolate leaves that are verticillate at the apex of branches, solitary and glabrous flowers, caducous bracts and bracteoles, stamens shorter than the corolla, anthers with prognathous thecae, and laterally connate tubules. The taxonomic similarities of the new species are discussed, and information about its distribution, habitat, and conservation is provided.

## Introduction

Approximately 70–75 species (POWO 2025) of the neotropical genus *Thibaudia* Ruiz & Pav. ex J.St.-Hil. are distributed from Honduras in Central America, south through the Andes Mountains of South America to central Bolivia, and eastward into Suriname and Guayanan Brazil (Luteyn 1996, Luteyn et al. 2009, Luteyn 2018). Its greatest diversity is found in Colombia (19 species/12 endemic, Pedraza-Peñalosa et al. 2016) and Ecuador (18 species/12 endemic, Luteyn 1996, León-Yánez et al. 2011). The revision of Luteyn (1996) for the genus in Ecuador provided an artificial key to the species in that country. In 2018, Luteyn described five new species from Peru and Bolivia, where ca. 15 species occur (13 endemic, Luteyn and Pedraza-Peñalosa unpubl.), and noted that there were also several other collections from that range that appeared to be new, but for which herbarium material was insufficient.

*Thibaudia* is difficult to circumscribe. For the most part, the species are distinct, but some of the characters that have been used traditionally to define the genus are also found in several other closely related genera, such as * Anthopterus, Themistoclesia *and *Cavendishia* (see, for example, Smith 1932, Smith 1936, Smith 1946, Macbride 1959, Luteyn 1996). Based on molecular data, the genus is polyphyletic (Kron et al. 2002, Powell and Kron 2003, Becker et al. 2024). Current molecular phylogenetic work indicates real problems of generic circumscription in neotropical Vaccinieae in general and that further generic realignments may be necessary.

The Cordillera del Cóndor is a mountain range along the Ecuador-Peru border, extending approximately 150 km. It is part of the sub-Andean cordilleras, a series of lower mountain ranges separated from the main Andes (Neill 2005, Ulloa and Neill 2006). Unlike the Andes, which are primarily of metamorphic and igneous origin, the Cordillera del Cóndor is composed mainly of sedimentary rocks, particularly sandstone and limestone (Rodríguez-Rodríguez et al. 2013). These geological differences contribute to the region’s unique edaphic conditions, particularly the nutrient-poor soils, which support distinctive vegetation types, including low, dense scrub-like forests (Neill 2005, Pérez et al. 2020, Clark and Neill 2023). The region receives precipitation from both the Pacific and Amazonian sides of the continent, leading to frequent, year-round rainfall (Almendáriz et al. 2014). These unique conditions have resulted in strong phytogeographic affinities with the Guiana Shield, as evidenced by numerous plant taxa with disjunct distributions between the two regions (Ulloa and Neill 2006, Rodríguez-Rodríguez et al. 2013, Zapata et al. 2021). This exceptional combination of factors has fostered high levels of plant endemism, with many species remaining undescribed (Jadán and Aguirre 2013, Pérez et al. 2020). During recent botanical explorations in the Shagmi area of the Cordillera del Cóndor, we discovered a new species of *Thibaudia*. Here, we describe and illustrate this species, providing detailed morphological and ecological information, as well as an assessment of its conservation status. Contrary to some views that fieldwork is no longer necessary, this study points out once again not only the richness of the flora in Ecuador but also, more importantly, the still desperate need for collecting and basic herbarium research.

## Materials and methods

Type specimens of all species of *Thibaudia* reported for Ecuador and northern Peru were reviewed from digital images through Global Plants on JSTOR (https://plants.jstor.org/) and GBIF (https://www.gbif.org). Moreover, the original descriptions of similar species (Smith 1952, Luteyn 1996) were reviewed and compared. The taxonomic key to the Ecuadorian species of *Thibaudia* by Luteyn (1996) was consulted to compare the new species to morphologically similar congeners. The new species was described in accordance with the botanical terminology of Beentje (2016). 

Measurements of the vegetative and floral parts were made from living material. Fresh flowers were preserved in 70% ethanol, 29% water, and 1% glycerol solution. Photos of dissected specimens were taken with a Panasonic FZ300 camera with a Raynox DCR-250 mm Super Macro lens. A distribution map was prepared with ArcGIS Desktop 10.3. The geographic coordinates of the specimen were omitted for conservation reasons; however, detailed information can be consulted in the herbarium voucher.

## Taxon treatments

### Thibaudia shagmiana

M.M. Jiménez, Luteyn & Darío García sp. nov.

CC01CF33-D5E8-58CE-8BB5-43E973D4ED6E

77366121-1

#### Materials

- **Type status:** Holotype. **Occurrence:** catalogNumber: HUTPL15454; recordNumber: M.M. Jiménez & D. Medina 2512; recordedBy: Jiménez, M.M.; occurrenceID: D942499F-BBFF-55E4-B250-EBB45ADDC5DC; **Taxon:** scientificName: Thibaudiashagmiana M.M. Jiménez, Luteyn & Darío García; **Location:** country: Ecuador; stateProvince: Zamora Chinchipe; locality: Cordillera de Shagmi, Cordillera del Cóndor flank, between El Pangui and Los Encuentros; verbatimElevation: 1523 m; **Event:** year: 2025; month: 1; day: 29; **Record Level:** institutionCode: HUTPL!

#### Description

**Figure 1.**
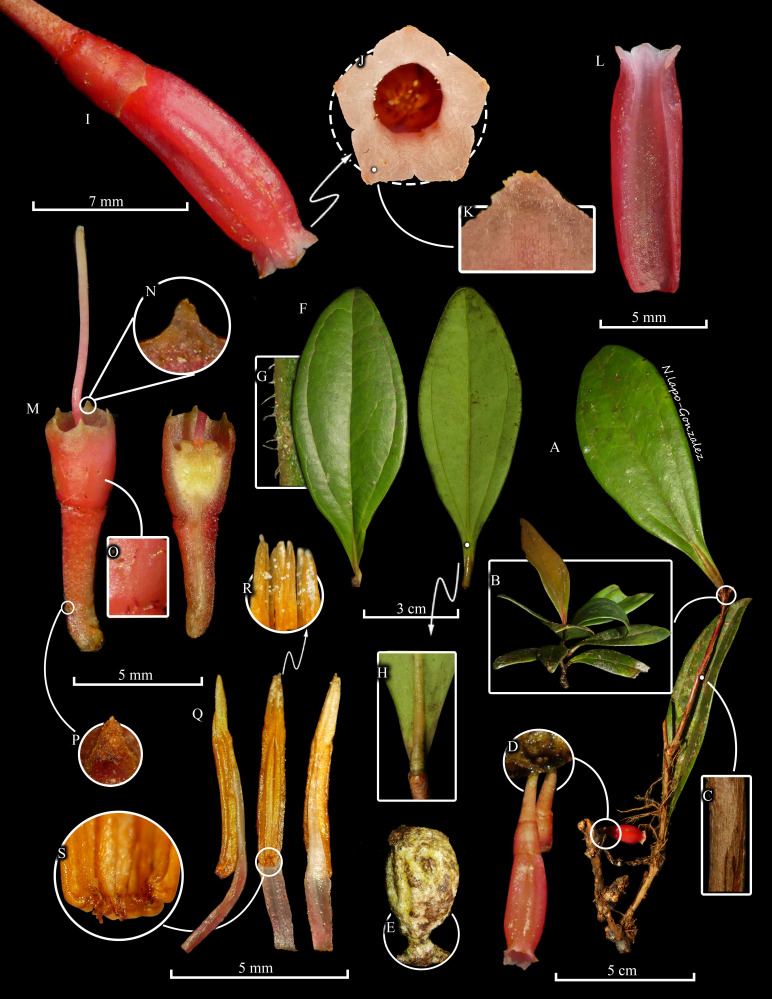
*Thibaudiashagmiana* M.M.Jiménez, Luteyn & Darío García. **A** Habit showing pseudoverticillate leaves (B), a close-up of a branch (C), inflorescence (D), and lignotuber (E). **F** Adaxial (right) and abaxial (left) views of leaves showing lateral margin (G) and adaxial base of leaf (H). **I **Corolla showing the ventral view of the corolla lobes (J) with a close-up of the surface (K). **L **Longitudinal section of calyx. **M **Calyx, pedicel and style with a close-up of lobes (N), hypanthium surface (O) and bracteole (P). **Q **Stamens with a close-up of apical slits of tubules (R) and the apex of thecae (S). Prepared by N. Lapo-González based on photographs by M.M. Jiménez of the type.

Plant erect, epiphytic shrub with fusiform lignotubers, 4.5–13.2 cm long, 4.0–9.4 in circumference; stems suberect to descending, terete to subterete, to 31.5 cm long, arising from a lignotuber, epidermis cracking longitudinally and exfoliating, striate after exfoliation, glabrous, brown in color, rooting when in contact with humus litter; axillary buds compressed, emerging ca. 1 mm above leaf node, bracts papyraceous, 1.9–4.7 mm long, long-triangular to subulate, glabrous. Leaves pseudoverticillate at apex of branch, suberect, subfalcate, sessile to subsessile; petiole (when present), complanate, 1.7–4.7 × 2.4–2.5 mm, glabrous, suffused with pink; blades thick-coriaceous, lanceolate to rarely elliptic, 2.4–10.9 × 1.3–4.2 cm, dull dark green adaxially, paler abaxially, centrally channelled and finely wrinkled adaxially, base cuneate to slightly attenuate, apex subacute, margin revolute, slightly incurved; venation conspicuously 3–5-plinerved from near base, midrib impressed and thickened in proximal 6–8 mm adaxially, raised and conspicuous abaxially, lateral nerves impressed adaxially and slightly raised abaxially, veinlets obscure, reticulate and anastomosing near margin. Inflorescence axillary, flowers solitary or 2-fasciculate, produced in leaf axils or on leafless branches; rachis short, 0.9–1.7 mm long × 1.1–1.2 mm diam., subterete, greenish with a touch of brown, covered by up to 4 caducous bracts, 0.8–0.9 mm long, these green but becoming brown with age; pedicel straight to slightly curved, subterete to obconical, subverrucose, 3.8–5.2 × 1.8–2.1 mm, magenta to scarlet with a brownish green base; floral bract 1, caducous, convex, 0.4-0.9 mm long, ovate to broadly ovate, apex obtuse, pale green becoming brown with age; bracteoles 2, located at base of pedicel, caducous, ovate, acute, convex, 1.1–1.4 × 1.0–1.1 mm, margin erose, whitish or brownish green with apex white, becoming brown with age. Flowers pentamerous, pendant, aestivation valvate; calyx articulate with pedicel, cupuliform overall, 3.9–4.4 × 2.8–3.6 mm, magenta to scarlet, paler at apex, glabrous but bearing scattered, short (ca. 0.2 mm long), brownish glandular fimbriae, hypanthium terete to subterete, slightly dilated to middle, rounded at base, 2.0–2.6 × 2.8–3.2 mm, limb cylindrical, 1.6–2.1 × 2.8–3.6 mm, lobes deltoid, apiculate, 0.4–0.5 × 0.8–0.9 mm, sinuses rounded; corolla carnose, bistratose, tubular, broadly and bluntly pentagonal (not angled or winged), narrowing to base, expanding slightly distally, glabrous, 11.1–14.0 mm long × 2.1 mm diam. at base, 3.0 mm diam. at throat, magenta to scarlet, lobes spreading, 0.8–1.3 × 1.5–2.0 mm, pinkish-white externally, internally glabrous, trigonous, dull, papillose. Stamens 10, shorter than corolla, equal with each other, 7.7–9.3 mm long; filaments equal, connate, 4.6–6.5 mm long, glabrous, pale pink; anthers 5.1–6.3 mm long overall, thecae 3.1–4.5 mm long, conspicuously papillose and prognathous at base, tubules 2, laterally connate, flattened, glabrous, 1.7–2.1 mm long, dehiscing by introrse apical slits ca. 1.4 mm long. Style 8.6–9.5 mm long, shorter than corolla, glabrous, white to pink or pink below middle, greenish at apex; stigma truncate; nectariferous disc annular. Fruit not seen. (Fig. 1).

#### Diagnosis

*Thibaudiashagmiana* is distinguished from other members of the genus by its combination of scrambling plant (to 30 cm) and lignotuberous with branches that root from nodes when in contact with mossy litter, lanceolate leaves that are pseudoverticillate at apex of branches, caducous bracteoles, solitary-flowered or 2-fasciculate inflorescences on short rachis, glabrous flowers produced in axils of lower leaves or on leafless branches, cupuliform calyx with a cylindrical limb, corolla with trigonous lobes, stamens ⅔ the corolla length, staminal filaments connate, prognathous thecae, laterally connate tubules, and a style that is shorter than the corolla.

#### Etymology

The new species is named after the Cordillera de Shagmi, a west-facing slope  of the Cordillera del Cóndor region in Zamora-Chinchipe province, Ecuador, where this species was found.

#### Distribution

**Figure 2.**
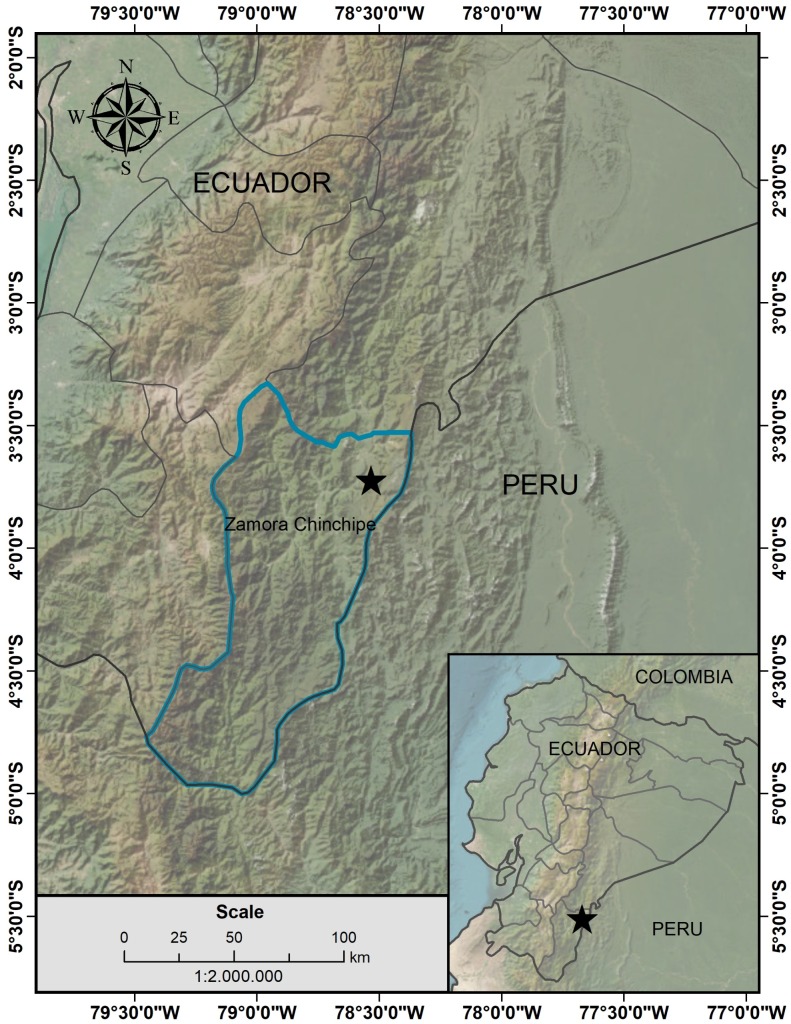
Geographic distribution of *Thibaudiashagmiana* in the southeastern region of Ecuador (prepared by H.X. Garzón-Suárez with ArcGIS).

*Thibaudiashagmiana* is currently known only from the type locality (around seven individuals recorded along a 230-m transect), within an Andean tepui (Neill et al. 2014) in the Shagmi sector, a mountainous chain located on the slopes of the Cordillera del Cóndor in Zamora Chinchipe province, southeastern Ecuador (Fig. 2). The tepui spans approximately 9000 m² and comprises a sandstone plateau at elevations ranging from 1400 to 1700 m a.s.l. (Vélez-Abarca et al. 2023).

#### Ecology

**Figure 3.**
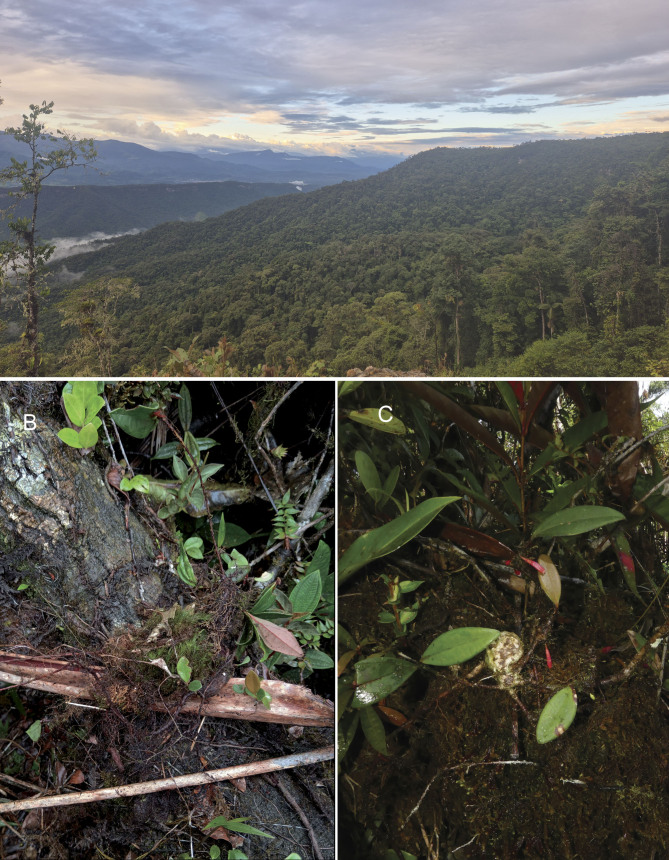
*Thibaudiashagmiana* in situ. **A** Habitat of the species. **B** An individual on a fallen tree branch. **C **Holotype of the species on a tree trunk.

According to the Ministerio del Ambiente del Ecuador (2013), the ecosystem where *Thibaudiashagmiana* is found corresponds to the Low Montane Evergreen Forest on Sandstone Plateaus of the Cordillera del Cóndor-Kutukú region (BsBa03). This area is characterized by a mean annual temperature of 20.4°C, and total annual precipitation of approximately 2148 mm. The climate is perhumid, with frequent fog and strong winds. The vegetation is generally low with scattered trees and extensive wooded shrub reaching a maximum height of 15 m (Fig. 3). The most common species include *Podocarpusoleifolius* D.Don, *Vochysiacondorensis* Huamantupa & D.A.Neill, *Scheffleraharmsii* J.F.Macbr., and *Magnoliayantzazana* F.Arroyo, along with an abundance of epiphytic plants, particularly among which are bryophytes and orchids.

#### Conservation

*Thibaudiashagmiana* is known only from the Shagmi sandstone plateau in the Cordillera del Cóndor, which is near El Pangui. The vegetation and soil conditions of this area are not suitable for cattle grazing or drystock pastures, which consequently protects the species' habitat from excessive deforestation. Only several individuals are known from the type locality. Regardless, this population is located within an actively operating mining concession, which is currently in operation. This activity may put the long-term conservation of the virgin habitat and its soil/aquifer chemistry at risk (*e.g*., an access road has just been opened that transects the habitat where this species is found). 

Applying conservation analysis with the georeferenced single locality, the calculated area of occupancy (AOO) is 4 km2. The extent of occurrence (EOO) has not been calculated due to the limited number of populations sighted. We recommend a conservation status of Critically Endangered (CR) according to the IUCN (2024) criteria B2ab(i,ii), C2a(i), and D1 is proposed here. The authors feel this assessment is accurate considering its presence outside of any protected area and the evident threat from the mining activity; this taxon is subject to higher levels of concern in the short term.

## Discussion

*Thibaudiashagmiana* belongs to the genus *Thibaudia*by having alternate leaves; pentamerous flowers with a tubular corolla containing ten stamens all of equal length; thecae that are as long as or half-length of tubules; and tubules that are fused laterally with each one provided of an elongate, strorse slit. These characteristics combined are morphological traits that currently represent genus *Thibaudia*(Luteyn and Pedraza-Peñalosa 2021). 

**Figure 4.**
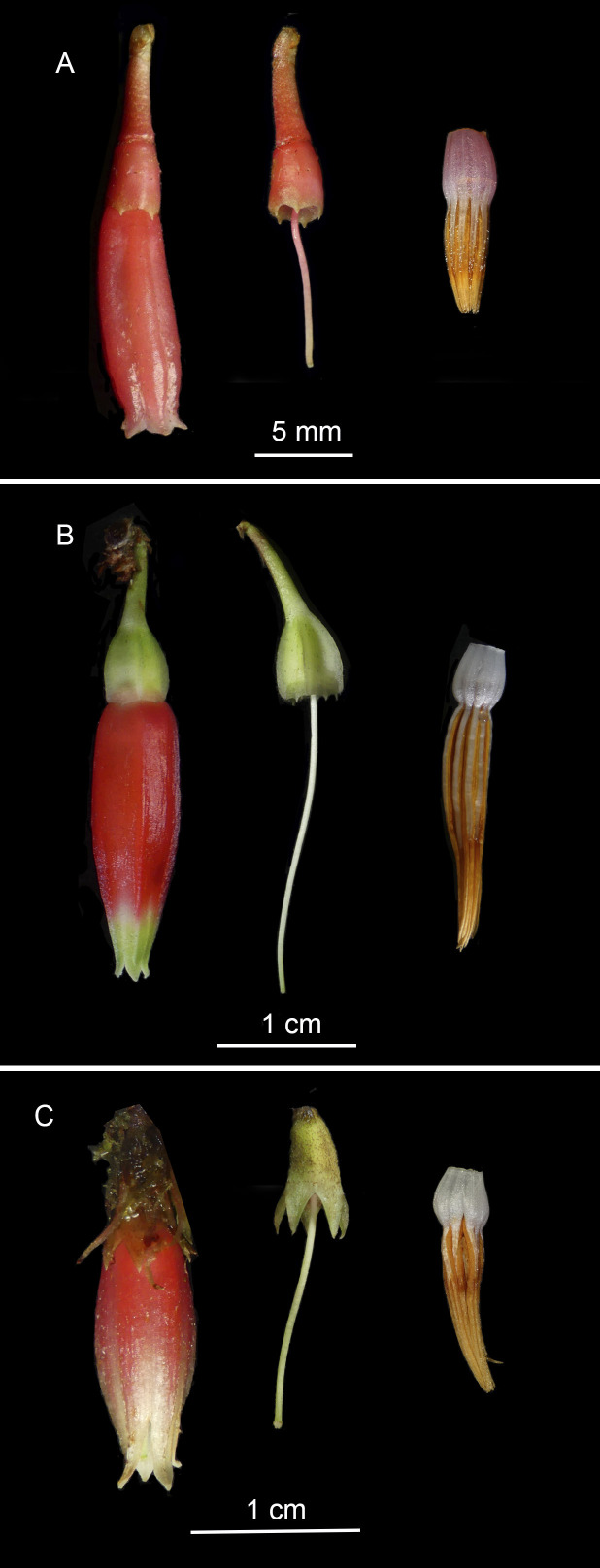
Floral comparison between related species. **A** *Thibaudiashagmiana*. **B** *T.lateriflora.* **C** *T.sessiliflora*.

This species has a trailing habit with rooting at nodes, leaves are elliptic to lanceolate, with inflorescences being either solitary or with few-flowered fascicles sometimes arising from leafless nodes, and anther tubules are short, making the new species most similar to *T.lateriflora* A.C.Sm. and *T.sessiliflora* (A.C.Sm.) Luteyn (Fig. 4). *Thibaudiashagmiana* can be distinguished from these two species by its conspicuously 3–5-plinerved leaves that are pseudoverticillately placed at apex of branches (vs. spirally arranged and 5–7-plinerved), calyx articulated with pedicels (vs. continuous), calyx terete to subterete (vs. angled or narrowly winged), corolla bluntly and broadly pentagonal (vs. angled or narrowly winged), stamens ⅔ the corolla length (vs. equaling the corolla), anther thecae prognathous at base (vs. rounded or mucronate), anther tubules proportionally shorter than the thecae and connate (vs. equal to or longer and distinct), and style shorter than the corolla (vs. equal to or exserted) as proposed by Smith (1952).

Another distinguishing feature of the new *Thibaudiashagmiana* is the developed lignotubers, whereas the nearest related species of *Thibaudia*are rhizomatous. Lignotubers are also known in *T. steyermarkii *A.C.Sm., which as a species differs in its pilose indumentum for its vegetative and floral anatomy. Furthermore, the leaves are ovate, and the calyx is concealed at base by numerous bracts (Luteyn 1996). Several species in the Ecuadorian Ericaceae, especially of the genera *Bejaria, Cavendishia, Ceratostema*, *Macleania* and *Vaccinium,* are also characterised by having lignotubers (Luteyn 1996, Luteyn 2002, D. García, personal observations), but these differ substantially in all vegetative and floral characteristics.

The distribution, habitat and ecology of *Thibaudiashagmiana* also provide further evidence for taxonomic segregation. The new species grows at an elevation of around 1500 m and is restricted to the lower montane forests on top of sandstone plateaus of the Cordillera del Cóndor in the northeast region of Zamora Chinchipe. According to Luteyn (1996), *T.lateriflora* is distributed at lower elevations from 500 up to 1400 m and restricted to the lowland and premontane forests of the provinces of Napo, Pastaza, and Morona Santiago in Ecuador, while *T.sessiliflora* grows at elevations from 500 to 1600 m in premontane, lower montane forests and rainforests further north in the province of Sucumbíos and to the south in Morona Santiago. Both species have their southernmost documented records in the Cordillera del Cutucú (another subrange north of the Cordillera del Cóndor).

## Supplementary Material

XML Treatment for Thibaudia shagmiana
